# Editorial: Endocrine aspects of gynaecological cancers

**DOI:** 10.3389/fendo.2024.1377378

**Published:** 2024-02-09

**Authors:** Bastian Czogalla, Maria Magdalena Montt-Guevara, Udo Jeschke

**Affiliations:** ^1^ Department of Obstetrics and Gynecology, LMU University Hospital, LMU Munich, Munich, Germany; ^2^ Division of Obstetrics and Gynecology, Department of Clinical and Experimental Medicine, University of Pisa, Pisa, Italy; ^3^ Department of Obstetrics and Gynecology, University Hospital Augsburg, Augsburg, Germany

**Keywords:** gynecological cancer, endocrinology, hormones, pathways, diagnostics, therapy

We are delighted to present this comprehensive Editorial for the Research Topic, “*Endocrine Aspects of Gynaecological Cancers*.” This Research Topic serves as a vital compendium at the intersection of endocrinology and gynaecological malignancies. These articles delve into the intricate interplay between hormonal factors and the development, progression, and management of these cancers, offering invaluable insights into this critical area of research and clinical practice.

## Understanding gynaecological cancers through an endocrine lens

Gynaecological cancers are a significant global healthcare challenge, impacting millions of women each year. This diverse group of malignancies encompasses cervical, ovarian, endometrial, vaginal, and vulvar cancer, presenting a complex puzzle for researchers and clinicians. The following articles within this Research Topic delve into various aspects of gynaecological cancers with a specific emphasis on endocrine factors, advancing our understanding of these diseases.

Guo et al. conducted a comprehensive review of Mendelian Randomization (MR) studies concerning endometrial cancer (EC). While observational studies have identified connections between modifiable environmental risk factors and EC, issues like unmeasured confounding and reverse causality have limited causal inferences. MR analysis, utilizing genetic variation independent of environmental factors, has provided insights into EC research and therapeutic potential. This review outlines MR analysis principles, summarizes EC-related MR studies, and explores links between various risk factors and EC. MR studies have identified associations of factors like type 2 diabetes, uterine fibroids, higher body mass index, and elevated hormone levels with increased EC risk, while later age at menarche and specific blood markers are associated with lower EC risk. Despite limitations, MR analysis remains valuable for investigating EC risk factors.

Turla et al. contributed a case report, systematic literature review, and pooled analysis on ovarian strumal carcinoid. This rare tumor, characterized by the coexistence of thyroid and carcinoid components, poses diagnostic and management challenges. Their study enhanced our understanding of this unique entity. Surgery was the primary therapy for most patients, but recurrence and metastasis were observed in a small subset. The study underscores the need for long-term follow-up, especially in cases with voluminous disease at diagnosis.

Gao et al. introduced a predictive nomogram for ovarian tumor malignancy using clinical markers. It involved 1,268 patients who underwent surgical removal of ovarian tumors. Analysis of various clinical markers, including post-menopausal status, BMI, HE4, CA125, ROMA index, disease course, PG-SGA score, ascites, and mass characteristics, identified significant variables for malignancy prediction. The resulting nomogram demonstrated superior predictive accuracy compared to conventional markers like CA125 and HE4, offering potential improvements in ovarian tumor diagnosis and management.

Du et al. explored a prognostic model for cervical cancer using ferroptosis-related gene expression. The model exhibited robust predictive capabilities, underlining its accuracy rooted in the tumor microenvironment. Notably, CA9 mRNA was prominently upregulated in cervical carcinoma tissues, suggesting its involvement in the disease. The study identified a ceRNA pair (CA9/ULBP2) potentially contributing to cervical cancer development, with hsa-miR-34a as a potential regulator. Moreover, the research uncovered miRNAs and drugs associated with these gene expressions, providing insights into the role of ferroptosis and potential therapies in cervical cancer.

Lu et al. investigated the potential role of elafin, a protein associated with immune infiltration, in predicting prognosis in ovarian cancer (OC). Higher elafin expression was associated with unfavorable prognoses, validated across different datasets. Importantly, elafin emerged as an independent risk factor for OC. Analyses also revealed links between elafin expression and immune-related pathways and immunotherapy responses, suggesting potential diagnostic and therapeutic implications.

Hu et al. conducted a study comparing small cell carcinoma of the ovary (SCCO) with high-grade serous ovarian cancer. SCCO is a rare, aggressive gynaecological cancer. SCCO patients were more likely to be diagnosed early, receive radiotherapy, and have larger tumors. SCCO had significantly worse survival rates than high-grade serous ovarian cancer. Prognostic models were developed for both cancer types, emphasizing the need for tailored SCCO management due to its unique characteristics.

Dahmani et al. examined the role of adrenal-derived 11-oxygenated androgens in EC. In a cohort of 272 postmenopausal EC cases, weak correlations were found between 11-oxygenated androgens and canonical androgens. Higher preoperative levels of free 11-ketoandrosterone were linked to an increased risk of recurrence, while postoperative levels of free 11β-hydroxyandrosterone were associated with worse recurrence and disease-free survival outcomes. These findings suggest that 11-oxygenated androgen metabolites could serve as potential prognostic markers for EC.

Zhao et al. used genome-wide association studies to investigate the impact of testosterone-related factors on gynecological diseases. It revealed that total and bioavailable testosterone were protective against OC and endometriosis but posed risks for EC and polycystic ovary syndrome (PCOS). Dehydroepiandrosterone sulfate protected against endometriosis and premature ovarian failure but increased risks for EC and PCOS. Sex hormone-binding globulin (SHBG) was protective against EC and PCOS. These findings imply causal links between serum testosterone levels and gynecological disorders, offering insights into their mechanisms.

## Conclusion: advancing knowledge, improving care

Gynaecological cancers continue to present a formidable challenge in healthcare. This Research Topic exemplifies the dedication and innovation of researchers worldwide in unraveling the complexities of these cancers through the lens of endocrinology. Each article contributes a piece to the puzzle, enhancing our understanding of the etiology, diagnosis, and treatment of these malignancies.

We extend our heartfelt appreciation to the authors for their valuable contributions, to the reviewers for their rigorous evaluation, and to the readers for their interest in this critical area of research. Together, we continue to push the boundaries of knowledge, with the ultimate goal of improving the lives of individuals affected by gynaecological cancers.

In closing, we invite you to explore the articles within this Research Topic. We believe that the insights presented here have the potential to transform our approach to the prevention, diagnosis, and treatment of gynaecological cancers. We look forward to witnessing the continued advancements in this field and the positive impact on patient care.

## Author contributions

BC: Writing – original draft. MM: Writing – review & editing. UJ: Writing – review & editing.

